# Application of lidar to assess the habitat selection of an endangered small mammal in an estuarine wetland environment

**DOI:** 10.1002/ece3.10894

**Published:** 2024-02-01

**Authors:** Jason S. Hagani, John Y. Takekawa, Shannon M. Skalos, Michael L. Casazza, Melissa K. Riley, Sarah A. Estrella, Laureen M. Barthman‐Thompson, Katie R. Smith, Kevin J. Buffington, Karen M. Thorne

**Affiliations:** ^1^ Suisun Resource Conservation District Suisun City California USA; ^2^ U.S. Geological Survey Western Ecological Research Center Dixon California USA; ^3^ California Department of Fish and Wildlife West Sacramento California USA; ^4^ California Department of Fish and Wildlife Fairfield California USA; ^5^ California Department of Fish and Wildlife Stockton California USA; ^6^ WRA, Inc. San Rafael California USA; ^7^ Department of Wildlife, Fish and Conservation Biology UC Davis Davis California USA; ^8^ U.S. Geological Survey Davis Field Station, University of California Davis Davis California USA

**Keywords:** remote sensing, salt marsh harvest mouse, small mammal, Suisun marsh, vegetation structure

## Abstract

Light detection and ranging (lidar) has emerged as a valuable tool for examining the fine‐scale characteristics of vegetation. However, lidar is rarely used to examine coastal wetland vegetation or the habitat selection of small mammals. Extensive anthropogenic modification has threatened the endemic species in the estuarine wetlands of the California coast, such as the endangered salt marsh harvest mouse (*Reithrodontomys raviventris*; SMHM). A better understanding of SMHM habitat selection could help managers better protect this species. We assessed the ability of airborne topographic lidar imagery in measuring the vegetation structure of SMHM habitats in a coastal wetland with a narrow range of vegetation heights. We also aimed to better understand the role of vegetation structure in habitat selection at different spatial scales. Habitat selection was modeled from data compiled from 15 small mammal trapping grids collected in the highly urbanized San Francisco Estuary in California, USA. Analyses were conducted at three spatial scales: microhabitat (25 m^2^), mesohabitat (2025 m^2^), and macrohabitat (~10,000 m^2^). A suite of structural covariates was derived from raw lidar data to examine vegetation complexity. We found that adding structural covariates to conventional habitat selection variables significantly improved our models. At the microhabitat scale in managed wetlands, SMHM preferred areas with denser and shorter vegetation and selected for proximity to levees and taller vegetation in tidal wetlands. At the mesohabitat scale, SMHM were associated with a lower percentage of bare ground and with pickleweed (*Salicornia pacifica*) presence. All covariates were insignificant at the macrohabitat scale. Our results suggest that SMHM preferentially selected microhabitats with access to tidal refugia and mesohabitats with consistent food sources. Our findings showed that lidar can contribute to improving our understanding of habitat selection of wildlife in coastal wetlands and help to guide future conservation of an endangered species.

## Graphical abstract

Light detection and ranging (lidar) has rarely been applied in wetland ecosystems or to study the habitat selection of small mammals. We demonstrate the efficacy of lidar in examining the role of vegetation structure in determining the habitat selection of the endangered salt marsh harvest mouse (*Reithrodontomys raviventris*) in a brackish wetland in California. We found that salt marsh harvest mice selected microhabitats with characteristics that provide refugia from tides and predators, such as taller and denser vegetation and proximity to levees. At the mesohabitat scale, salt marsh harvest mice were associated with a lower percentage of bare ground and pickleweed (*Salicornia pacifica*) presence. Preserving higher elevation wetland habitat features such as levees and supporting a heterogeneous mixture of shorter and taller vegetation types will benefit salt marsh harvest mouse populations.

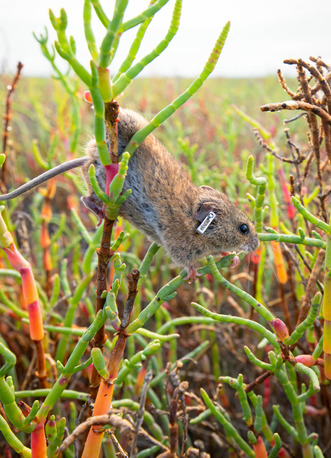

## 1 INTRODUCTION

**FIGURE 1:**
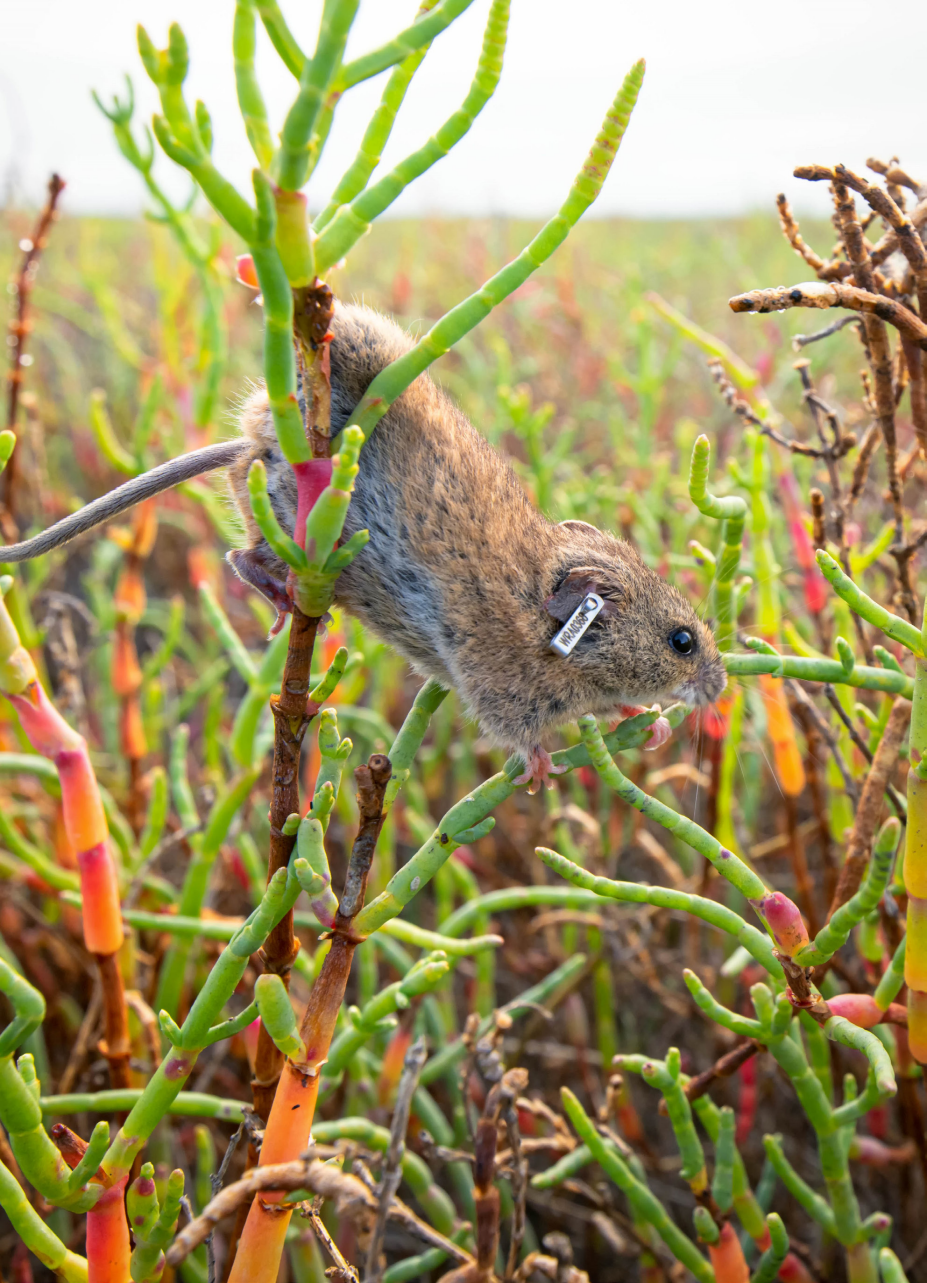
An ear‐tagged salt marsh harvest mouse (*Reithrodontomys raviventris*) climbing through pickleweed (*Salicornia pacifica*) habitat (photo: Marisa Ishimatsu).

Over the last few decades, extensive development and human activity have threatened coastal wetland ecosystems worldwide (Casazza et al., 2021; Davidson, 2014; Li et al., 2018; Marcot et al., 2020). It is estimated that as much as 87% of global coastal wetlands have been lost due to diking, filling, and other anthropogenic activities (Davidson, 2014; Smith et al., 2020). Wetlands are crucial ecosystems for a variety of native plant and wildlife species, including halophytes, waterfowl, fish, and small rodents (Marcot et al., 2020; Moyle et al., 2014). The salt marsh harvest mouse (*Reithrodontomys raviventris*, hereafter SMHM; Figure 1) is one such species endemic to the highly urbanized (Nichols et al., 1986) coastal wetlands of the San Francisco Estuary. SMHM are fully confined to coastal wetlands and directly adjacent habitats (Smith et al., 2018). Habitat loss, degradation, and fragmentation have resulted in the listing of SMHM as endangered at both the state and federal level (CNDDB, 2023; Shellhammer et al., 1982; USFWS, 1984, 2013; Whitaker & NatureServe, 2018). The continued preservation of SMHM habitat is therefore a priority for conservation practitioners in the region (USFWS, 2013). Currently, a substantial proportion of the remaining SMHM population resides in Suisun Marsh – part of the San Francisco Estuary and one of the largest contiguous brackish marshes in North America (Smith et al., 2018; Sustaita et al., 2011). However, the San Francisco Estuary has been particularly vulnerable to anthropogenic influence; <10% of its historic tidal wetlands remain today (Bias & Morrison, 1999; Smith et al., 2014; Williams & Faber, 2001). A comprehensive understanding of SMHM ecology and behavior can help wildlife practitioners conserve the species, especially in the face of impending sea‐level rise due to climate change which is already negatively impacting habitat (Moyle et al., 2014; Smith et al., 2018; Spencer et al., 2016; Thorne et al., 2014, 2018).

Habitat selection is widely considered to be an important aspect of the ecology of a species (Marcot et al., 2020; Mayor et al., 2009; Morris, 2003; Padgett‐Flohr & Isakson, 2003; Vierling et al., 2008). Elevation and vegetation structure have been shown to be crucial considerations of habitat selection for a variety of species, including birds (Cody, 1981; Guyot et al., 2017; Jedlikowski et al., 2016; Tsao et al., 2009), small mammals (Jaime‐González et al., 2017; Klinger et al., 2015), and ungulates (Ewald et al., 2014). Determining the environmental characteristics which make some aspects of the wetland landscape more valuable than others as potential habitat to SMHM will support its conservation. There have been several studies on the movement and habitat selection of the SMHM (Bias & Morrison, 2006; Shellhammer et al., 1982; Smith et al., 2020; Sustaita et al., 2011); however, these studies have not quantified vegetation structure in detail because it is difficult to quantify using standard field methods. SMHM are hypothesized to rely upon taller plants as tidal refugia and protection from predators (Bias & Morrison, 2006; Smith et al., 2014; Sustaita et al., 2011), while utilizing shorter plants for food; therefore, although it has not been studied in depth, vegetation structure within their potential habitat may be a crucial factor in their habitat selection.

Airborne topographic light detection and ranging (lidar) has emerged as a valuable tool for monitoring ecological phenomena and examining the three‐dimensional components of a landscape (Davies & Asner, 2014; Simonson et al., 2014). Its applications are wide‐ranging; lidar has been employed by ecologists to assess animal species diversity (Davies & Asner, 2014; Simonson et al., 2014) landscape structure and health (Lim et al., 2003; Richardson & Moskal, 2011), and the habitat selection of wildlife. However, use of lidar in habitat selection studies has been primarily limited to avian species in forest canopy (Goetz et al., 2010; Hagar et al., 2020; Vierling et al., 2008) and forest mammals (Ewald et al., 2014; Jaime‐González et al., 2017). While lidar has been particularly valuable for studying the habitat selection of species for which there is large variation in the vegetation height structure, the tool has rarely been used in wetland ecosystems that have a relatively limited range of ground and vegetation heights (Koma et al., 2020). Also, it has been rarely used to examine the habitat selection of small mammals (Jaime‐González et al., 2017). Prior to widespread availability of lidar, measurements of vegetation structure were often compiled as field‐based metrics collected by hand (Jaime‐González et al., 2017; Koma et al., 2020). These measurements can be somewhat coarse or subjective and collecting the data may be costly and time‐consuming (Freeman et al., 2022; Jaime‐González et al., 2017; Vierling et al., 2008). Therefore, lidar may allow users to quantify vegetation structure in habitats at a much finer scale while covering a much broader extent (Hagar et al., 2020; Vierling et al., 2008).

This study aimed to examine the characteristics of vegetation structure that may provide nuanced insight into the habitat selection of SMHM. We also took advantage of the opportunity to evaluate the ability of lidar to assess habitat selection in a coastal wetland ecosystem, because SMHM likely rely on a three‐dimensional landscape for many crucial ecological functions (Bias & Morrison, 2006; Smith et al., 2014). We used data compiled from small mammal field surveys conducted throughout Suisun Marsh in conjunction with a suite of lidar‐derived covariates to determine the characteristics of preferred SMHM habitat. Because habitat selection is often made at different spatial scales (Guyot et al., 2017; Jedlikowski et al., 2016; Johnson, 1980; Mayor et al., 2009), we examined the relative importance of structural and non‐structural characteristics of the wetland in predicting SMHM habitat at three spatial scales. We hypothesized that SMHM would preferentially select habitats with vegetation structure characteristics that provide access to refugia and food sources and that lidar‐derived covariates would improve understanding of their habitat preferences over traditional habitat selection models.

## 2 METHODS

### 2.1 Study site

**FIGURE 2:**
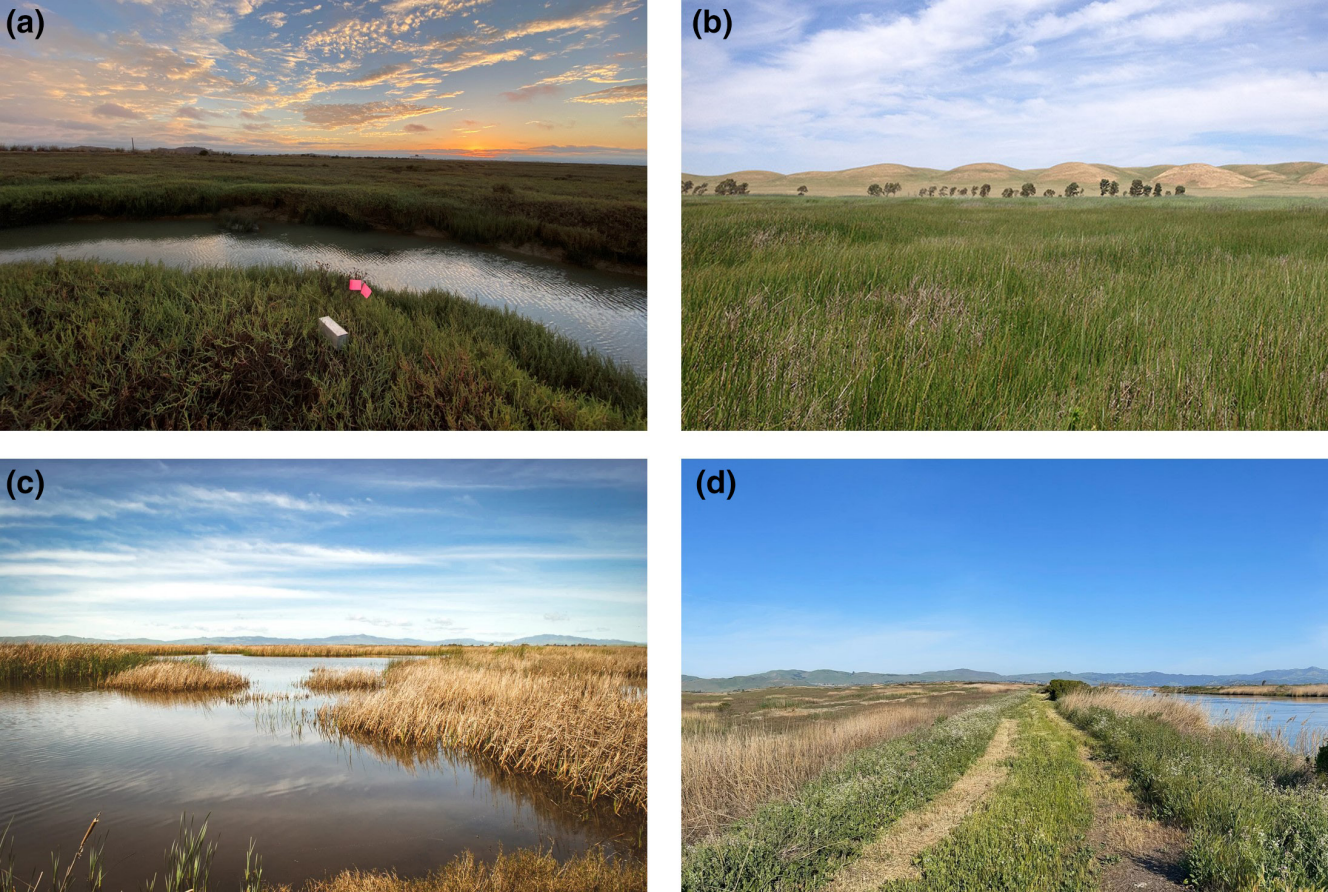
(a) A small mammal trap placed in the field, (b) an upland area dominated by grassland (photo: San Francisco Bay National Estuarine Research Reserve), (c) a tidal wetland subjected to the influence of semidiurnal tides (photo: Westervelt Ecological Services), (d) a managed wetland surrounded by levees on which water levels are controlled.

We examined the habitat selection of SMHM in Suisun Marsh in the San Francisco Estuary in northern California, USA. We considered habitat selection as an individual's use of certain areas in the ecosystem proportionately more than their availability (Mayor et al., 2009). Suisun Marsh (38°08′28.1″ N, 122°00′43.6″ W) is a 46,950‐ha region divided by a mix of public, private, and nonprofit landowners. The wetlands are either seasonal managed wetlands surrounded by levees with water infrastructure (flood and drain gates) controlling the water levels primarily for waterfowl hunting, or tidal wetlands open to the influence of the mixed semidiurnal daily tides resulting in twice‐daily high and low tidal inundation (Figure 2). Additional non‐wetland, upland areas are interspersed throughout the marsh and consist of grassland and pasture. These upland areas are often found adjacent to wetlands.

Many characteristics of Suisun Marsh are unlike any other portion of the SMHM geographic range (USFWS, 2013). The mosaic of wetlands and habitat types in Suisun Marsh, each with varying water management type (actively managed and tidal) and plant communities, provides a diversity of unique habitat patches that SMHM may select. In addition, the brackish water of Suisun Marsh promotes greater vegetation diversity than other portions of the SMHM geographic range (Jones et al., 2021). Most wetlands in the San Francisco Estuary outside Suisun Marsh where SMHM are found are oligohaline marshes and primarily composed of pickleweed (*Salicornia pacifica*; Padgett‐Flohr & Isakson, 2003). Therefore, it is likely that the habitat selection of SMHM in Suisun Marsh differs from those in other parts of the San Francisco Estuary.

### 2.2 Trapping surveys

**FIGURE 3:**
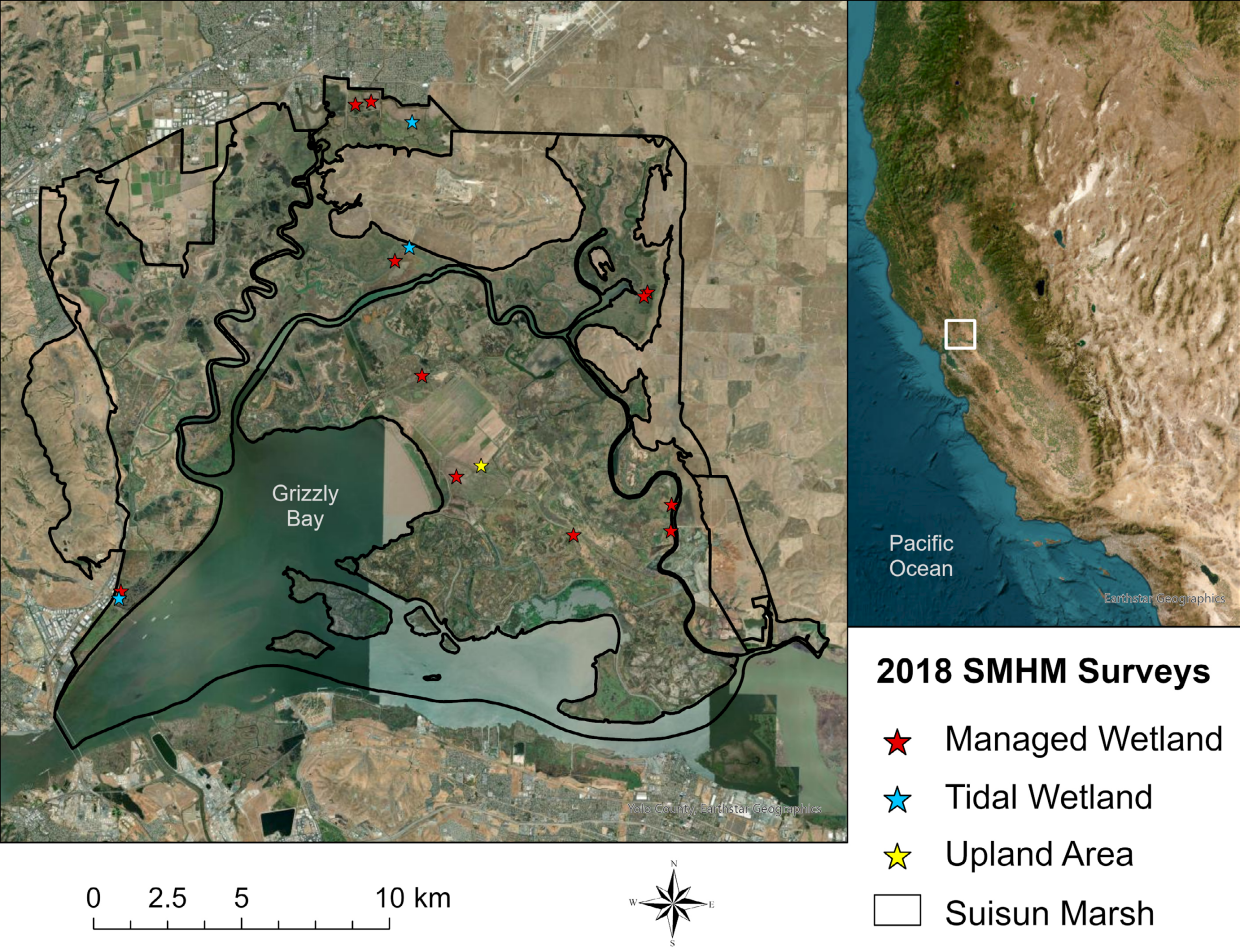
Location of the 15 small mammal surveys conducted in Suisun Marsh used in this study. Red stars indicate the survey was conducted in a managed wetland (surrounded by levees with water infrastructure controlling the water levels), blue stars in a tidal wetland (open to the influence of the mixed semidiurnal daily tides resulting in twice‐daily high and low tides), and yellow stars in an upland area (grassland areas adjacent to the wetlands). Suisun Marsh is located within the white square in the inset map of California on the right. SMHM, salt marsh harvest mouse.

We compiled data from small mammal surveys that were conducted at trapping grids on 15 sites throughout Suisun Marsh (Figure 3). SMHM were trapped at 11 managed wetlands, 3 tidal wetlands, and 1 upland area. Trapping grid arrays varied from 49 to 100 traps, and surveys were conducted for either three or four trap nights (Table 1). An accessible area was used to tether the grid, after which uniform spacing was used to place traps in a configuration that best represented the broader landscape. While the number of traps and survey duration varied across sites depending on the size and shape of grids, the methodology employed at each remained consistent. Traps were located 10–15 m apart and were sampled in the early morning. Each trap location was measured using a Garmin Oregon 650 t or a Garmin eTrex 10 handheld GPS, which have a maximum accuracy of 3–5 m in ideal conditions. Due to the lack of tree cover in Suisun Marsh and because locations were averages taken by a stationary user, we considered our GPS locations to be accurate within this range. All surveys were conducted during the summer (June and July) of the year in which we obtained the lidar image of the region. SMHM were identified in the field by trained wildlife biologists with expertise in SMHM identification. A regression model (Sustaita et al., 2018) was applied using tail length, body length, and tail diameter which assisted in confirming species identification along with surveyor expertise, as SMHM can be difficult to distinguish from the congeneric western harvest mouse (*R. megalotis*; Statham et al., 2016; Statham et al., 2021). All SMHM were captured in 2018 and handled by permitted biologists operating under incidental take permit TE‐020548‐14 (USGS), scientific collecting permit SC‐005749 (USGS), and Institutional Animal Care & Use Committee (IACUC) permits #19806 and #21118 (UCD). All SMHM surveys used in this study were conducted under the cooperative agreement between California Department of Fish and Wildlife (CDFW) and the U.S. Fish and Wildlife Service (USFWS).

**TABLE 1:** Metadata for the 15 small mammal surveys used in this study.

Site name	Grid area (ha)	Habitat type	Traps	Orientation	Survey duration (days)
Area 9	1.64	Tidal	100	Fit to wetland	4
Arnold A	0.81	Managed	50	5 × 10	4
Arnold B	0.81	Managed	50	5 × 10	4
Crescent Unit	1.82	Managed	100	10 × 10	4
Field 14P	0.81	Upland^a^	49	7 × 7	3
Goodyear Managed	1.22	Managed	60	6 × 10	3
Goodyear Tidal	1.22	Tidal	60	6 × 10	3
Hill Slough 4	0.81	Managed	50	5 × 10	4
Hill Slough 4A	0.81	Managed	50	5 × 10	4
Joice Managed	1.22	Managed	60	6 × 10	3
Joice Tidal	1.22	Tidal	60	6 × 10	3
Pond 1	0.81	Managed	49	5 × 10^b^	3
Pond 2	0.81	Managed	49	5 × 10^b^	3
Pond 15	0.81	Managed	49	7 × 7	3
Pond 20	0.81	Managed	49	7 × 7	3

*Note*: Habitat type was managed wetland (surrounded by levees with water infrastructure controlling the water levels), tidal wetland (open to the influence of the mixed semidiurnal daily tides resulting in twice‐daily high and low tides), or upland (grassland areas adjacent to the wetlands).

^a^ Upland field surrounded by wetlands.

^b^ One row of nine traps.

### 2.3 Lidar data

Airborne topographic lidar data collection was contracted by the California Department of Water Resources (DWR) to Towill, Inc. (Concord, CA, USA). These discrete return lidar data were collected over a two‐day period in September 2018 with a Teledyne/Optech Orion 300 sensor. The survey achieved an average density of eight total lidar returns per m^2^ with an accuracy of 7‐cm root mean squared error (RMSE); these parameters classify the collection as QL1 data. The lidar strips were then processed and calibrated using Optech's LMS software suite and data from seven Continuously Operating Reference Stations (CORS) located throughout the Suisun Marsh area.

### 2.4 Habitat structure variables

**FIGURE 4:**
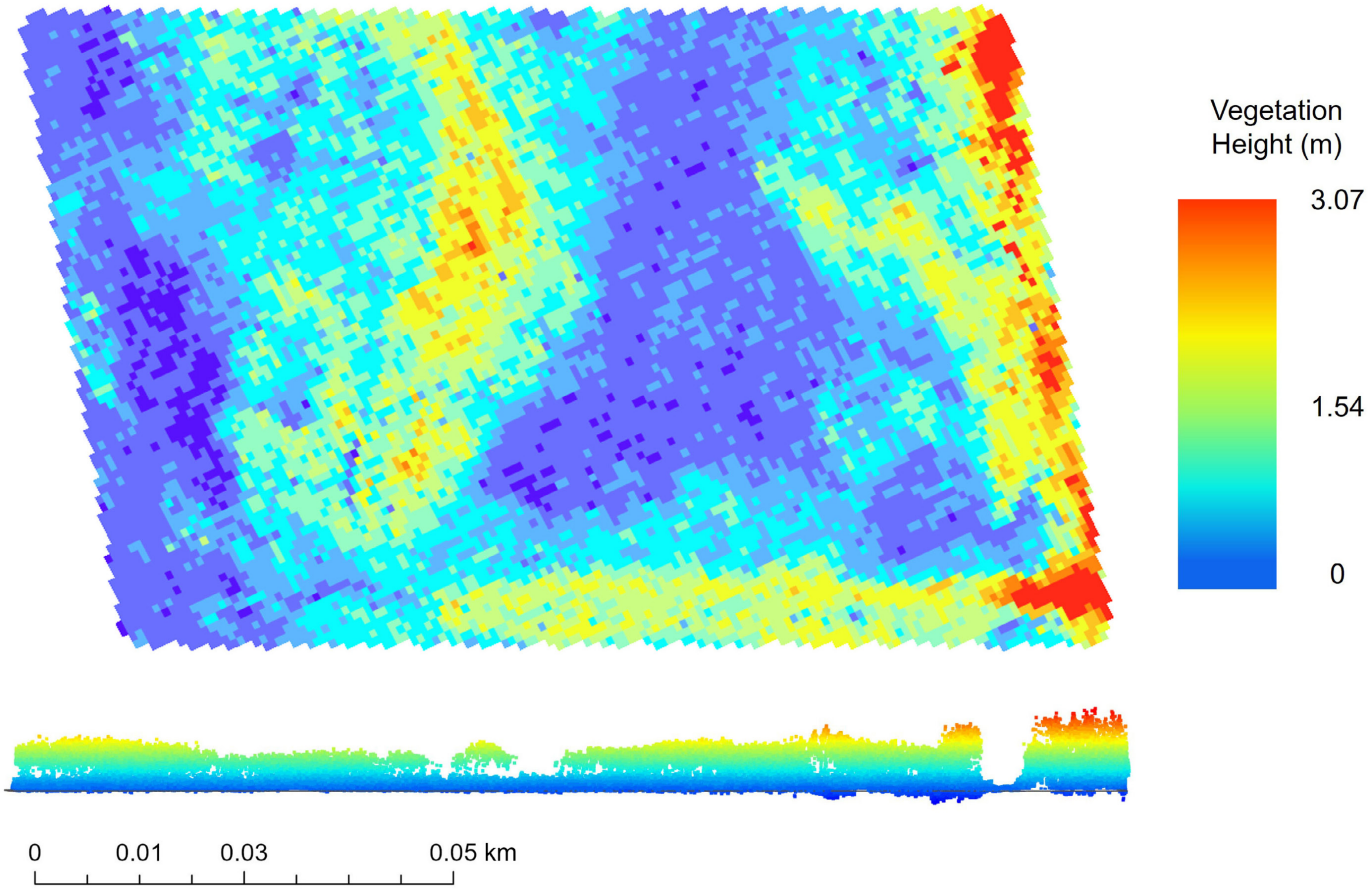
Example lidar point cloud at the Joice Managed survey grid in Suisun Marsh. Top image shows the birds‐eye view of the grid as a 1 m^2^ raster and the bottom view shows the point cloud from the side. Vegetation heights are displayed in colors, with blue/green representing shorter vegetation and warmer colors representing taller vegetation. Maximum vegetation height within this grid was 3.07 m. For better visualization, vegetation heights in the bottom view have been scaled 10×.

We derived a set of variables which describe vegetation structure (Table 2) from the raw lidar point cloud. These variables were chosen because of their previous use in assessing the vegetation complexity of rodent habitat (Jaime‐González et al., 2017) or to examine ecosystem structure more generally (Bakx et al., 2019; Koma et al., 2020). Many of the variables are related to the *Z*‐axis of a lidar return (“*Z*”; vegetation height) or the percentage of vegetation height distribution (“*P*”). For each trapping grid, the relevant lidar file(s) were isolated, merged into a single file, and clipped to the grid using the LAStools software suite (Isenburg, 2014) in ArcGIS Pro (ESRI, 2019; Figure 4). A 3‐m buffer was established around the perimeter of each trapping grid to ensure edge data were properly captured. In R Studio Version 1.2.5033 (R Core Team, 2021; R Studio Team, 2021), using the package “lidR” (Roussel et al., 2020; Roussel & Auty, 2022), the clipped lidar file was normalized to set all points delineating the ground surface to have a height of 0 m. We used a digital elevation model (DEM) corrected with a modification of the Lidar Elevation Adjustment with NDVI (LEAN) technique (Buffington et al., 2019) to normalize the point cloud, rather than the raw lidar data itself. Surface elevation heights as estimated by this DEM were subtracted from the “*Z*” value of the raw lidar data, resulting in a measurement of vegetation height. Because SMHM inhabit areas characterized by relatively short vegetation, and because aerial lidar may not penetrate the dense vegetation canopy of tidal wetlands, the DEM was corrected to ensure the accuracy of our normalized lidar data (Buffington et al., 2016). The typical vertical accuracy of aerial lidar is 15–25 cm, RMSE; the corrected DEM produced an accuracy of 7 cm, RMSE (Buffington et al., 2019). The suite of lidar‐derived metrics was then extracted from the normalized lidar file (Table 2).

**TABLE 2:** Lidar‐derived, three‐dimensional habitat structure variables.

Metric	Definition	Description
Zmean^a^ ^,^ ^b^	Mean vegetation height	Mean vegetation height
Zmax^b^	Maximum vegetation height	Maximum vegetation height
ZSD^a^ ^,^ ^b^ ^,^ ^c^	Standard deviation of vegetation height	Describes the complexity of the surrounding vegetation
Zskew^b^	Skewness of vegetation height	A skew value closer to 0 suggests a normal distribution of vegetation height
Zkurt^b^	Kurtosis of vegetation height	A high kurtosis can suggest outliers in the distribution of vegetation height
PZ>Zmean^a^ ^,^ ^c^	Percentage of returns above mean vegetation height	A lower percentage implies the existence of outlier vegetation above mean height
PZ>X^a^ ^,^ ^c^	Percentage of returns above X m	Describes the percentage of vegetation taller than X (0.25, 0.50, 0.75) m
ZQ5 to ZQ95^c^	Q quantile of height distribution	The vegetation height at each 5% quantile (ZQ5, ZQ10, etc.). ZQ100 (100%) is equivalent to the maximum height; ZQ50 is equivalent to the median
ZC1 to ZC9^c^	Cumulative percentage of return of the *N*th bin	Divides the height distribution into 10 equal parts, each part (ZPC1, ZPC2, etc.) describing the percent of observations found below it
%Bare^b^ ^,^ ^c^	Percentage of returns classified as “ground”	Describes the percentage of lidar returns classified as bare ground (no vegetation)

*Note*: Variables were derived from a point cloud that had been normalized (ground points set to zero) using a modified digital elevation model (Buffington et al., 2019). “*Z*” refers to *z*‐axis (vegetation height), “*P*” refers to percentage, “*Q*” refers to quantile, and “*C*” refers to cumulative. These variables were derived at the microhabitat (25 m^2^), mesohabitat (2025 m^2^), and macrohabitat (trapping grid size, ~10,000 m^2^) scale.

^a^ Jaime‐González et al. (2017).

^b^ Bakx et al. (2019).

^c^ Koma et al. (2020).

**FIGURE 5:**
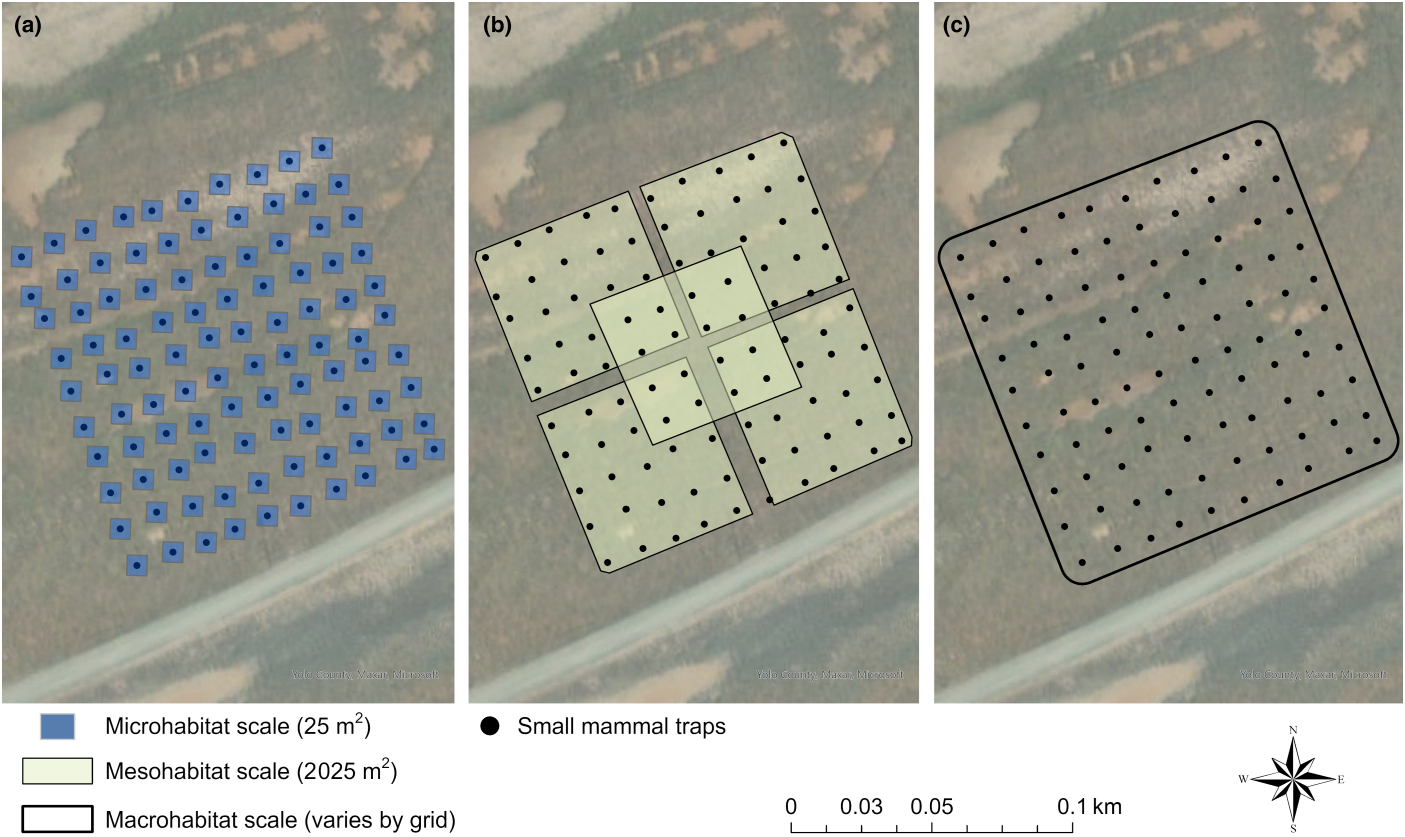
Visualization of the three spatial scales at which the habitat selection of salt marsh harvest mice (*Reithrodontomys raviventris*) was analyzed at the Crescent Unit trapping grid. The three spatial scales were (a) microhabitat (trap level; blue squares; 25 m^2^), (b) mesohabitat (home range level; yellow squares; 2025 m^2^), and (c) macrohabitat (trapping grid level; black outline; size varies by grid). The location of small mammal traps is represented by the black dots.

We examined habitat selection at three scales: microhabitat (trap level; 25 m^2^), mesohabitat (home‐range level; 2025 m^2^; Bias & Morrison, 1999), and macrohabitat (trapping grid level; ~10,000 m^2^; Figure 5; Table 1). For the microhabitat scale, we selected 5 m as the minimum resolution possible given the accuracy of our GNSS receivers for a pixel size of 25 m^2^. All lidar points collected within each spatial resolution were used to calculate habitat structure variables. Habitat selection is often made at various spatial scales (Guyot et al., 2017; Jedlikowski et al., 2016; Johnson, 1980), and the fine‐scale resolution at which lidar is collected allows for flexibility in analyses. Therefore, our assessment of SMHM habitat selection at these three scales allowed us to test the efficacy of lidar in assessing important habitat and structural characteristics at different levels.

### 2.5 BASE covariates

Some standard habitat variables have been shown to help characterize habitats preferred by SMHM in earlier studies. These variables include ground surface elevation (“Elev”), distance to nearest levee (“LDist”), distance to nearest urban area (“UDist”), and dominant vegetation species (“Veg”; Bias & Morrison, 2006; Basson, 2009; Tsao et al., 2009; Sustaita et al., 2011; Marcot et al., 2020). Ground surface elevation was extracted from the corrected DEM (Buffington et al., 2019), and we used the Euclidean Distance tool in ArcGIS Pro to create a 3‐m raster describing the distance of every pixel to the nearest levee (Unpubl. data, SRCD) or urban area. These raster files were reprojected to match the coordinate system of the raw lidar data (NAD83 California Zone 2, in feet; EPSG:2226). Lastly, we included the dominant plant species from vegetation data collected in the field during each of the surveys used in this study. This variable was recorded in the field during the duration of the trapping survey and identifies the single most prevalent plant species within five meters surrounding each trap (see Smith et al., 2020). Previous research on SMHM habitat selection in other parts of the San Francisco Estuary has included similar covariates such as distance to nearest permanent water and distance to nearest road (Marcot et al., 2020). However, because most “roads” in Suisun Marsh are on levees, which border most permanent water sources, we expected our distance to levee and urban area covariates to be sufficient.

The use of these variables may dominate the results of models seeking to explain more subtle elements of habitat selection including habitat structure. To look more closely at the selection for specific elements of habitat structure, we followed the methods described in Žydelis et al. (2006) and forced the four standard variables into all our multivariate models grouped as a single “BASE” covariate (Kemp et al., 2023; Morin et al., 2020). By condensing these four covariates into a single group, we reduced the number of possible candidate models and allowed for a focused assessment of the relative importance of lidar‐derived structure covariates (Morin et al., 2020).

### 2.6 Statistical models

Before model creation, and at each spatial scale, we tested for correlation among covariates by calculating Pearson's coefficients in R. The BASE covariate was separated, and each covariate was assessed individually for this test. Covariate combinations with coefficients >0.70 were examined, and the variable hypothesized to be less relevant to SMHM habitat selection was removed (Taylor, 1990). We then standardized all continuous covariates with *z*‐score normalization to set the mean of each to 0 and the standard deviation to 1 (De Knegt et al., 2010). This process ensured that variable coefficients produced by our models would be directly comparable.

To examine the habitat selection of SMHM, we fit generalized linear models (GLMs) in R. To account for differences in both the survey duration and number of traps in a grid, we used capture efficiency (CE) as our response variable. CE can be defined as:

(1) $$\mathrm{CE}=100\left(\frac{\text{Total number of unique mice captured}}{\text{Number of traps}\times \text{Number of sample days}}\right)$$

Our selection of “unique” captures ensured that a recapture of an individual mouse was not included in our models multiple times. In instances where an individual was captured more than once, the first capture occasion was used in analyses. We compared candidate models with the Akaike Information Criterion (AIC). Models with an AIC difference >2.0 were considered statistically inequivalent with lower AICs indicating greater parsimony (Anderson & Burnham, 2004; Harrison et al., 2018). For models with comparable AIC values (ΔAIC <2.0), the model with the fewest parameters was selected. As additional metrics of model performance, we calculated R‐squared values and Akaike weights for each candidate model (Lok et al., 2012) and used the sum of Akaike weights to assess the relative importance of individual model covariates (Giam & Olden, 2015). A null model including no predictor covariates was included at each spatial scale; however, this null model was never selected and therefore excluded from our results.

#### 2.6.1 Microhabitat models

At the microhabitat scale, we generated models for all traps as well as comparing those in tidal wetlands, managed wetlands, and upland separately. Because each habitat type is subjected to varying levels of inundation and water control, we expected the vegetation requirements (and thus habitat selection) of SMHM to differ within each. We generated models with the BASE covariate and habitat structure variables separately, and the BASE and structure covariates in a single model. We then used backward stepwise regression (Zhang, 2016) to identify the most parsimonious model that included both BASE and habitat structure covariates. Nonsignificant terms were then iteratively removed from the full model until the most parsimonious model was identified.

#### 2.6.2 Mesohabitat models

Previous research has estimated SMHM home‐range sizes in salt marshes of San Pablo Bay immediately west of Suisun Marsh to be 2000 m^2^ (0.2 ha) in size (Bias & Morrison, 1999), and we used this home‐range size to generate potential SMHM home ranges in each trapping grid. Applying this home‐range size, we created representative 45 × 45 m home range polygons (2025 m^2^) and located them in the corners and center of each trapping grid. We calculated an average for each habitat structure variable and the BASE covariate within each home range. All traps within a home range were used to derive an estimate of CE, and these resulted in 3–5 replicates depending on the shape of the grid. Some traps were resampled more than once in 11 of the grids, but 75% of the total were only sampled once. However, we felt the resampling with replacement was justified for some grids for representation of average habitat structure variables, the BASE covariate, and CE within the home ranges (Fieberg et al., 2020). As with our microhabitat models, we generated models with only the BASE covariate, only habitat structure variables, and the BASE and structure covariates combined. We determined the most parsimonious BASE and structure model with backward stepwise regression. We also included habitat type (tidal wetland, managed wetland, upland) as a categorical covariate.

#### 2.6.3 Macrohabitat models

We ran univariate models on each non‐correlated covariate. BASE covariates were separated and assessed individually at this spatial scale. Habitat structure and BASE covariates were averaged within each grid. Habitat type was included as a categorical covariate. To reconcile the small sample size, we used corrected AIC (AICc; Cavanaugh, 1997) to compare univariate models.

## 3 RESULTS

### 3.1 Summary statistics of SMHM surveys

The number of unique SMHM captured at a survey site ranged from 0 to 60, with an average of 19 (±16 SD). Six unique SMHM were captured in the one upland site, 70 across three tidal sites, and 210 across 11 managed sites. CE was highest in Field 15 (managed wetland; 26.53), lowest in Arnold A and Arnold B (managed wetlands, 0.00), and averaged 9.4 (±6.94). CE was highest in managed wetlands (9.78), followed by tidal wetlands (9.21) then upland area (4.08).

Mean vegetation height was highest in the Joice Tidal survey site (0.52 m), lowest in Hill Slough 4 (0.01 m), and averaged 0.23 m (±0.14). Maximum vegetation height was highest in Joice Tidal (3.17 m), lowest in Crescent Unit (0.58 m), and averaged 1.60 m (±0.84). Hill Slough 4 had the highest percentage of bare ground (75.68%) and Joice Tidal the lowest (28.87%). Bare ground percent averaged 51.37% across all grids (±12.23).

At traps at which SMHM were captured, maximum vegetation height averaged 0.57 m (±0.46), mean vegetation height averaged 0.25 m (±0.24), and percent bare ground averaged 51.76% (±21.22). At traps where no mice were captured, maximum vegetation height averaged 0.53 m (±0.40), mean vegetation height averaged 0.23 m (±0.20), and percent bare ground averaged 54.56% (±21.90).

### 3.2 Microhabitat models

Combining traps from all habitat types into one model reduced its predictive power, with the best model for all traps explaining just 11% of the variation in the data (Table 3). In comparison, the best model for tidal wetland traps explained 23% of variation, 15% for managed wetland traps, and 32% for upland traps. Based on AIC values, models for both wetland types (tidal wetland, managed wetland) significantly improved by combining the BASE covariate and habitat structure variables (Table 3). For our upland site, the model including only habitat structure variables was statistically comparable with our best model combining BASE and structure covariates.

**TABLE 3:** Comparison of candidate models at the trap‐level (25 m^2^; “microhabitat”) spatial scale.

Ecosystem type	Model	AIC	ΔAIC	*R* ^2^	Akaike weight
All	Full	7869.7	3.4	.11	0.15
	BASE only	7881.5	15.2	.08	0.00
	Habitat Structure only	7909.6	43.3	.03	0.00
	Best (BASE + Zmax + PZ > 0.25 + ZQ5 + ZPC5 + ZPC9 + %Bare)	7866.3	0	.11	0.85
Tidal Wetlands	Full	1976.4	7.1	.24	0.03
	BASE only	1973.9	4.6	.17	0.09
	Habitat Structure only	2000.8	31.5	.06	0.00
	Best (BASE + Zmax + ZQ5 + ZPC1 + ZPC6 + ZPC9 + %Bare)	1969.3	0	.23	0.88
Managed Wetlands	Full	5454.1	6.0	.15	0.05
	BASE only	5471.7	23.6	.10	0.00
	Habitat Structure only	5484.8	36.7	.06	0.00
	Best (BASE + %Bare + Zskew + ZPC1 + ZPC4)	5448.1	0	.15	0.95
Upland Areas	Full	417.9	4.2	.38	0.06
	BASE only	417.2	3.5	.15	0.08
	Habitat Structure only	413.7	0	.30	0.47
	Best (BASE + PZ > Zmean + ZPC1 + ZPC8 + ZQ5)	414.0	0.3	.32	0.40

*Note*: We ran models with all uncorrelated covariates (“full”), just the BASE covariate (“BASE only”), just habitat structure variables (“Habitat Structure only”), and with the most parsimonious combination of covariates (“Best”). The BASE covariate includes ground surface elevation, distance to nearest levee, distance to nearest urban area, and dominant vegetation species. For each habitat type, the covariates included in the best model are listed in parentheses. As metrics of model performance and/or model comparison, we found AIC, *R* ^2^, and Akaike weights. A null model was run for each analysis but never selected and was excluded from the results.

Across all traps, SMHM captures were significantly associated with a higher Zmax (*p* = .02), lower PZ > 0.25 (*p* = .01), ZQ5 (*p* < .01), and ZPC5 (*p* < .01), as well as saltmarsh aster (*Symphyotrichum subulatum*; *p* = .02) and baltic rush (*Juncus balticus*; *p* = .04) dominance (Table 4). While not statistically significant, %Bare (*p* = .06) and pickleweed dominance (*p* = .07) were strongly associated with SMHM captures.

**TABLE 4:** Outputs for the most parsimonious models combining the BASE covariate and habitat structure variables at the microhabitat scale (trap‐level; 25 m^2^).

Variable	Estimate	SE	*t*‐value	*p*‐value	*w*(*i*)
**All traps (*R*** ^**2**^ **= .11)**					
Intercept	2.17	9.28	0.23	.82	NA
Zmax	3.32	1.39	2.38	.02*	0.83
PZ > 0.25	−2.64	1.01	−2.62	.01*	0.87
ZQ5	−3.42	1.19	−2.88	<.01*	0.93
ZPC5	−4.18	1.14	−3.67	<.01*	0.97
ZPC9	1.29	0.87	1.48	.14	0.48
%Bare	−2.03	1.09	−1.88	.06	0.69
Elev	−1.02	1.23	−0.83	.41	0.30
LDist	1.85	0.94	1.97	.05*	0.60
UDist	−0.76	1.10	−0.69	.49	0.31
*Symphyotrichum subulatum*	26.80	11.83	2.27	.02*	1.00
*Atriplex prostrata*	15.82	10.78	1.47	.14	1.00
Bare Ground	10.04	10.05	1.00	.32	1.00
*Bromus diandrus*	1.67	11.80	0.14	.89	1.00
*Distichlis spicata*	4.14	9.39	0.44	.66	1.00
*Frankenia salina*	10.50	9.93	1.06	.29	1.00
*Juncus balticus*	19.96	9.88	2.02	.04*	1.00
*Phragmites australis*	9.69	10.58	0.92	.36	1.00
*Salicornia pacifica*	16.76	9.33	1.80	.07	1.00
*Schoenoplectus acutus*	9.15	11.78	0.78	.44	1.00
*Schoenoplectus americanus*	10.17	9.72	1.05	.30	1.00
*Sesuvium verrucosum*	0.20	12.12	0.02	.99	1.00
*Typha* spp.	−2.27	11.60	−0.20	.85	1.00
*Xanthium strumarium*	2.41	13.84	0.17	.86	1.00
**Tidal Wetlands (*R*** ^**2**^ **= .24)**					
Intercept	23.76	8.13	2.92	<.01*	NA
Zmax	8.78	3.11	2.83	.01*	0.65
ZQ5	−4.36	3.06	−1.43	.15	0.36
ZPC1	−4.75	1.79	−2.65	.01*	0.73
ZPC6	−5.66	2.67	−2.12	.03*	0.45
ZPC9	5.06	1.92	2.63	.01*	0.72
%Bare	4.31	2.26	1.91	.06	0.41
Elev	−1.79	1.57	−0.75	.45	0.35
LDist	−5.13	2.24	−2.29	.02*	0.66
UDist	−4.41	2.14	−2.06	.04*	0.67
*Atriplex prostrata*	−31.21	22.07	−1.41	.16	0.88
*Distichlis spicata*	−15.97	9.14	−1.75	.08	0.88
*Frankenia salina*	−23.60	15.59	−1.51	.13	0.88
*Juncus balticus*	2.73	9.07	0.30	.76	0.88
*Salicornia pacifica*	−3.17	8.55	−0.37	.71	0.88
*Schoenoplectus acutus*	−25.11	16.34	−1.54	.13	0.88
*Schoenoplectus americanus*	−4.98	8.77	−0.57	.57	0.88
*Typha* spp.	−17.11	11.21	−1.52	.13	0.88
**Managed Wetlands (*R*** ^**2**^ **= .15)**					
Intercept	5.62	9.22	0.61	.54	NA
PZ > 0.25	−3.26	1.40	−2.33	.02*	0.93
ZQ5	−2.24	1.14	−1.96	.05	0.74
ZPC2	2.05	1.40	1.47	.14	0.56
ZPC5	−3.63	1.16	−3.12	<.01*	0.94
%Bare	−3.99	1.20	−3.32	<.01*	0.98
Elev	−1.97	1.30	−1.51	.13	0.59
LDist	3.32	0.97	3.41	<.01*	0.99
UDist	−0.57	1.42	−0.40	.69	0.32
*Atriplex prostrata*	12.10	10.86	1.11	.27	1.00
Bare Ground	5.52	9.97	0.55	.58	1.00
*Bromus diandrus*	−4.33	11.69	−0.37	.71	1.00
*Distichlis spicata*	1.77	9.37	0.19	.85	1.00
*Frankenia salina*	5.38	10.50	0.51	.61	1.00
*Juncus balticus*	9.34	10.36	0.90	.37	1.00
*Phragmites australis*	7.39	10.45	0.71	.48	1.00
*Salicornia pacifica*	12.32	9.28	1.33	.18	1.00
*Schoenoplectus acutus*	17.60	11.78	1.49	.14	1.00
*Schoenoplectus americanus*	2.08	10.90	0.19	.85	1.00
*Sesuvium verrucosum*	−4.00	11.96	−0.33	.74	1.00
*Xanthium strumarium*	−0.29	13.62	−0.02	.98	1.00
**Upland Areas (*R*** ^**2**^ **= .32)**					
Intercept	−1.19	5.32	−0.22	.82	NA
PZ > Zmean	6.23	2.77	2.25	.03*	0.78
ZPC1	5.63	3.46	1.63	.11	0.38
ZPC8	−4.45	2.43	−1.83	.07	0.60
ZQ5	4.97	2.93	1.70	.10	0.50
Elev	1.85	2.55	0.73	.47	0.31
UDist	−0.66	5.09	−0.13	.90	0.35
LDist	−1.65	5.46	−0.30	.76	0.45
*Frankenia salina*	13.79	6.92	1.99	.05	0.52
*Salicornia pacifica*	7.17	6.84	1.05	.30	0.52

*Note*: Models were generated for all traps combined and for each habitat type (managed wetland, tidal wetland, upland area) separately. Variables that were significant at the *p* < .05 level are marked with an asterisk.

**FIGURE 6:**
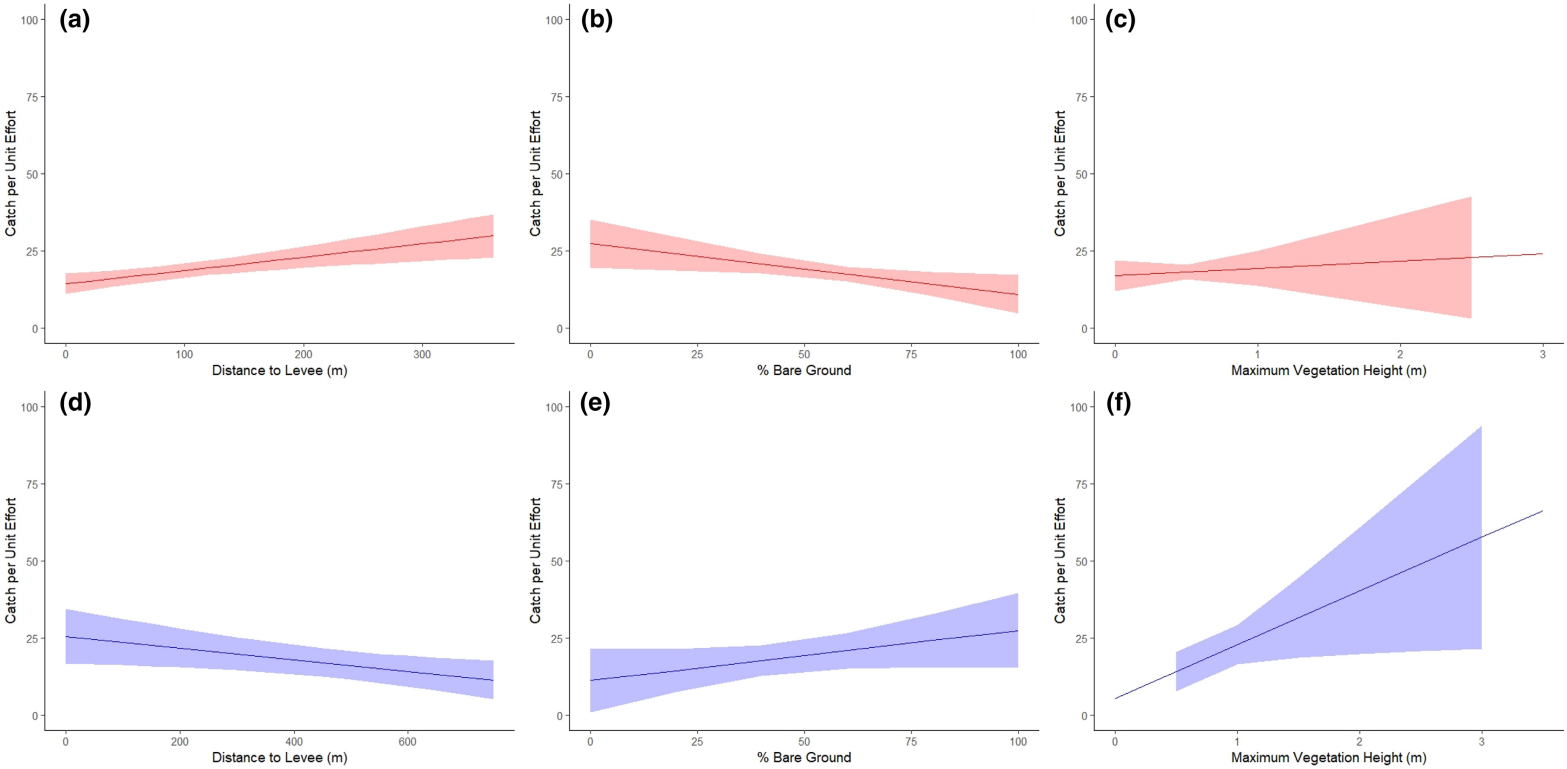
Partial effects plots showing the relationship between salt marsh harvest mouse (*Reithrodontomys raviventris*) catch per unit effort (CE) and important covariates in managed wetlands (a–c; red) and tidal wetlands (d–f; blue). We show the relationship between CE and distance to levees (a, d; “LDist), percent bare ground (b, e; “%Bare”), and maximum vegetation height (c, f; “Zmax”). These partial effects plots were derived from our full models at the microhabitat scale that included all lidar habitat structure variables and BASE covariates.

In tidal wetlands, SMHM were significantly associated with traps with a higher Zmax (*p* = .01; Figure 6) and ZPC9 (*p* = .01) and lower ZPC1 (*p* = .01) and ZPC6 (*p* = .03; Table 4). SMHM were also found closer to levees (*p* = .02) and urban areas (*p* = .04). SMHM captures tended towards microhabitats with a greater proportion of bare ground (*p* = .06) and where common tule (*Schoenoplectus acutus*; *p* = .13) and cattail (*Typha* spp.; *p* = .13) were the dominant plant species, although not significantly.

In managed wetlands, SMHM were associated with traps with a significantly lower PZ > 0.25 (*p* = .02), ZPC5 (*p* < .01), and %Bare (*p* < .01; Table 4; Figure 6). SMHM also preferentially selected traps further from levees (*p* < .01). PZ > Zmean (*p* = .03) was the only statistically significant covariate in our best upland area model, with SMHM preferentially found in areas with a greater proportion of vegetation above the mean vegetation height.

### 3.3 Mesohabitat models

Zmean, Zskew, ZQ5, ZPC2, ZPC5, ZPC9, and %Bare were found to be uncorrelated habitat structure variables and were included in models alongside the BASE covariate. Our full model produced an AIC of 404.9 (*R* ^2^ = .55). The BASE‐only model yielded an AIC of 414.0 (*R* ^2^ = .28); the structure‐only model 414.2 (*R* ^2^ = .22). The most parsimonious model combining BASE and habitat structure covariates significantly improved the results (AIC = 397.0, *R* ^2^ = .52). This best model included %Bare, PZ > Zmean, Zmean, ZPC2, and Zskew, in addition to the BASE covariate.

Mouse captures at the mesohabitat scale were significantly and negatively associated with %Bare (*p* = .01), ZPC2 (*p* = .01), UDist (*p* = .05), and multiple vegetation types (Table 5). SMHM captures were significantly (*p* = .04) and positively associated with areas where pickleweed was the dominant vegetation species. Mouse captures were also negatively related to Zmean, but not significantly (*p* = .09; Table 5). Based on the sum of Akaike weights, Veg (*p* = .97), ZPC2 (*p* = .94), and %Bare (*p* = .91) were the most important individual covariates; Elev (*p* = .38), PZ > Zmean (*p* = .58), and Zskew (*p* = .59) were the least.

**TABLE 5:** Model outputs for the most parsimonious model which included both habitat structure variables and the BASE covariates at the mesohabitat (2025 m^2^) spatial scale.

Variable	Estimate	SE	*t*‐value	*p*‐value	Akaike weight
Intercept	28.96	7.2	4.02	<.01	NA
%Bare	−5.24	1.77	−2.97	.01*	0.91
PZ > Zmean	2.21	1.51	1.49	.15	0.58
Zmean	−3.87	2.22	−1.75	.09	0.64
ZC2	−4.66	1.69	−2.76	.01*	0.94
Zskew	1.82	1.34	1.36	.18	0.59
Elev	−1.52	2.41	−0.63	.53	0.38
LDist	−0.19	0.84	−0.23	.86	0.11
UDist	−4.49	2.18	−2.07	.05*	0.78
*Atriplex prostrata*	−9.87	10.61	−0.93	.36	0.97
*Distichlis spicata*	−24.38	7.45	−3.27	<.01*	0.97
*Juncus balticus*	−7.22	9.54	−0.76	.45	0.97
*Phragmites australis*	−6.96	8.85	−0.79	.44	0.97
*Salicornia pacifica*	15.78	7.61	−2.07	.04*	0.97
*Schoenoplectus acutus*	−13.88	10.41	−1.33	.19	0.97
*Schoenoplectus americanus*	−20.47	7.72	−2.65	.01*	0.97

*Note*: Variables that were significant at the *p* < .05 level are marked with an asterisk.

### 3.4 Macrohabitat models

We found Zmean, Zskew, PZ > Zmean, ZPC2, ZPC5, ZPC9, and %Bare to be uncorrelated habitat structure covariates and therefore were included in models at this spatial scale. None of our univariate models produced statistically significant results. The model which included habitat type yielded the highest *R* ^2^ value (.12). PZ > Zmean (0.11) and ZPC2 (0.12) were the most important variables based on the sum of Akaike weights.

## 4 DISCUSSION

### 4.1 Lidar efficacy and value in wetlands

Airborne topographic lidar is a well‐established tool for researching the habitat selection of wildlife, but its use has been primarily reserved for avian species or forest‐dwelling animals (Ewald et al., 2014; Goetz et al., 2010; Hagar et al., 2020; Jaime‐González et al., 2017; Vierling et al., 2008). Few studies have attempted to employ lidar to assess structural characteristics of wetlands or ecosystems with a focus on habitat selection. Our results demonstrate that even for species that reside in ecosystems without a wide range of vegetation heights, lidar can reveal nuances in the structural complexity of the habitats. This is particularly important when wetland restoration or enhancement projects are done in the estuary. Widespread tidal wetland restoration is ongoing in the San Francisco Estuary (Callaway et al., 2011) and planned throughout Suisun Marsh (US DOI, 2013) with over 2000 ha under consideration (Goals Project, 1999). This restoration is planned to benefit native fish (Brown, 2003), California Ridgway's Rail (R*allus obsoletus obsoletus*), and SMHM (Goals Project, 1999). Applying lidar to better understand habitat preferences of SMHM could assist in the design and implementation of wetland restoration projects that aid in the recovery of the species (US DOI, 2013).

At both the microhabitat and the mesohabitat scale, models containing only the classic BASE landscape covariates or only habitat structure variables performed significantly worse than models that combined the two, suggesting that both the landscape covariates and habitat structure variables from lidar provided important insights into SMHM habitat selection. Our best model at the mesohabitat spatial scale, incorporating both the BASE covariate and habitat structure variables, explained 52% of the variation in the data. In comparison, similar models examining the habitat selection of wood mice (*Apodemus sylvaticus*) in a Mediterranean high‐mountain pine forest found that their best models including both lidar‐ and field‐derived covariates explained 30% of variance (Jaime‐González et al., 2017). At the microhabitat scale, adding habitat structure variables to the BASE covariate also improved our models significantly across habitat types. Although we only sampled a single upland study area, habitat structure variables improved the variance explained by the BASE‐only model from 15% to 32%.

Lidar can provide a potentially cheaper and less labor‐intensive alternative to field data collection while covering a larger geographic extent (Jaime‐González et al., 2017). The fine‐scale resolution at which data were collected with lidar for this study (8 points per m^2^) also presents flexibility in analysis and the opportunity to examine landscape characteristics at a variety of scales. Airborne topographic lidar may not penetrate dense vegetation which creates difficulties in examining features below the vegetation canopy (Buffington et al., 2016). Here, <1% of the point cloud captured in our study sites were second lidar returns suggesting that only the tops of vegetation were being measured. For animals such as SMHM which may utilize understory, thatch, and litter for cover, travel, and denning (Fisler, 1965; Marcot et al., 2020; Shellhammer et al., 1982), the inability to penetrate dense vegetation hinders the ability to fully examine habitat selection. Terrestrial lidar may reconcile this shortcoming and has been shown to accurately characterize litter in previous research (Loudermilk et al., 2009; Rowell & Seielstad, 2012). Similarly, lidar shot by unmanned aerial systems (UAS) can collect hundreds of returns per square meter (Pricope et al., 2022) and provide more accurate vegetative detail in wetland ecosystems.

### 4.2 Habitat selection of SMHM

Our analysis of lidar data revealed important characteristics of vegetation structure in assessing the habitat selection of SMHM at the microhabitat scale. Our results provide evidence to support the importance of vegetation heterogeneity in SMHM habitat. Across all sites, and in managed wetlands in particular, SMHM captures at the microhabitat scale were positively correlated with the standard deviation of vegetation height. No other habitat structure variables were significant in managed wetland sites, suggesting that SMHM are selecting microhabitats with a higher variance in vegetation heights without showing explicit preference for shorter or taller vegetation. Earlier research has hypothesized that SMHM utilize taller vegetation for both refugia from inundation at higher tides (tidal wetlands) or seasonal flooding (managed wetlands) and for cover from predators (Fisler, 1965; Marcot et al., 2020; Shellhammer et al., 1982; Smith et al., 2018). They rely on shorter vegetation such as grasses and pickleweed, as well as over 40 other plant and invertebrate species (Aylward et al., 2022; Smith & Kelt, 2019) for food. Microhabitats represent short‐term landscape use (Morris, 1987), but variation in heights of the vegetation may provide value for SMHM over longer periods.

In tidal wetlands, taller vegetation was the primary component of preferred SMHM microhabitat. SMHM selected areas with a higher maximum vegetation height and were associated with taller vegetation species like common tule and cattail, although that association was not significant. Similarly, SMHM captures were associated with a lower cumulative percentage in the first (ZPC1) and sixth vegetation bin (ZPC6), suggesting that a greater proportion of shorter vegetation within microhabitats is not preferred. Surprisingly, SMHM preferred areas with a higher bare ground percentage, potentially further highlighting the importance of refugia in their habitats. As tidal wetlands are subjected to daily inundation and more extreme flooding events, vegetation refugia may be more critical especially in areas with more bare ground.

In addition to taller vegetation, SMHM selected areas closer to levees in tidal wetland microhabitats. While levees are primarily found on managed wetlands, most tidal wetlands and upland areas in Suisun Marsh are also bordered by similar high‐elevation structures. Levees, roads, and other higher‐elevation areas may serve as refugia outside of core home ranges in the event of extreme flooding, while taller vegetation within the marsh plain acts as a consistent source of refugia habitat. While the influence of tides on SMHM movements is still not well known, previous research has suggested that SMHM are tolerant of levees and roads near their habitats (Marcot et al., 2020), may regularly cross levees (Bias & Morrison, 1999), or even use levees to avoid flooded areas (Hulst et al., 2001). Species that use similar habitats such as California black rails (*Laterallus jamaicensis coturniculus*; Tsao et al., 2009) have been shown to rely on levees for refugia as well. High water has also been shown to increase the hunting activity of avian predators in tidal wetlands (Thorne et al., 2019), increasing the value of refugia for predator avoidance.

In contrast, SMHM in managed wetlands selected for microhabitats with a lower proportion of vegetation taller than 0.25 m and a lower percentage of bare ground suggesting a preference for areas with shorter, denser vegetation. Taller vegetation may be necessary for inundation refugia, but dense vegetation may provide better protection from predation (Kotler, 1984; Thompson & Gese, 2013), and SMHM were also found in areas farther from levees. In managed wetlands, where water levels do not fluctuate daily, SMHM may not rely on elevated levees as regularly compared to tidal wetlands, though there may be seasonal differences that were not examined in this study.

Models generated at the mesohabitat scale performed the strongest with our most parsimonious model explaining 52% of the variation in the data. These results suggest that the home‐range scale may be the most important spatial level at which SMHM select their habitats. SMHM preferentially selected mesohabitat with a lower bare ground percentage indicating that abundance of vegetation is an important characteristic of their preferred habitat. This finding aligned with success criteria for wetland restoration, which set the requirements for providing SMHM habitat and often include greater percent vegetation cover (USFWS, 1981, 2013). Other important structural covariates at the mesohabitat scale provided evidence that shorter vegetation comprised the majority of selected habitats. SMHM were similarly associated with pickleweed presence which served as one of the most significant sources of food for the species (Aylward et al., 2022; Smith & Kelt, 2019). Our overall results suggested that microhabitats may offer SMHM temporary resources such as refugia from predation and tides, while mesohabitats offer abundant food to support individuals and populations in the longer term. Neither habitat structure variables nor BASE covariates were significant at the macrohabitat scale, and the grid scale may be too large to capture how individual SMHM select features in the landscape.

### 4.3 Future directions

Management of endangered species such as the SMHM is of great importance for resource managers in the San Francisco Estuary. With increasing threats of climate change and sea‐level rise (Craft et al., 2008; Elmilady et al., 2019; Knowles, 2010; Thorne et al., 2018), protecting SMHM habitats has never been more crucial. In Suisun Marsh, climate change is projected to raise sea levels, therefore lowering relative tidal wetland elevations, by 0.15–0.61 m by the end of the century (Takekawa et al., 2013; Thorne et al., 2018), likely placing many SMHM habitats at risk. Loss of coastal wetland habitats from sea‐level rise has been documented for other wetland species (Hunter et al., 2017; Nuse et al., 2015; Rosencranz et al., 2019) with wetland restoration activities proposed as a tool to combat loss (Veloz et al., 2013). While SMHM are now known to inhabit both tidal and diked wetlands at similar densities (Shellhammer et al., 1982; Smith et al., 2020; Sustaita et al., 2011), their affinity towards both wetland types puts a large proportion of the population at risk under sea‐level rise scenarios (Stralberg et al., 2011). Flooding and sea‐level rise also are expected to increase avian predation of tidal wetland wildlife, further threatening the survival of species like the SMHM (Thorne et al., 2019). Our findings suggest that preserving higher‐elevation wetland habitat features such as levees and supporting a heterogeneous mixture of shorter and taller vegetation types will benefit SMHM populations. These findings are consistent with current recommendations which focus on maintaining pickleweed presence, access to adjacent high‐marsh transition zones for refugia, and a mixture of short and tall vegetation species (USFWS, 2013). Continuing to prioritize habitats that provide the criteria described in this study could benefit SMHM conservation, especially in the face of climate change.

## AUTHOR CONTRIBUTIONS

**Jason S. Hagani:** Conceptualization (equal); formal analysis (equal); visualization (equal); writing – original draft (equal); writing – review and editing (equal). **John Y. Takekawa:** Conceptualization (equal); funding acquisition (equal); project administration (equal); writing – original draft (equal); writing – review and editing (equal). **Shannon M. Skalos:** Investigation (equal); writing – review and editing (equal). **Michael L. Casazza:** Investigation (equal); writing – review and editing (equal). **Melissa K. Riley:** Funding acquisition (equal); investigation (equal); writing – review and editing (equal). **Sarah A. Estrella:** Funding acquisition (equal); investigation (equal); writing – review and editing (equal). **Laureen M. Barthman‐Thompson:** Funding acquisition (equal); investigation (equal); writing – review and editing (equal). **Katie R. Smith:** Funding acquisition (equal); investigation (equal); writing – review and editing (equal). **Kevin J. Buffington:** Investigation (equal); writing – review and editing (equal). **Karen M. Thorne:** Investigation (equal); writing – review and editing (equal).

## FUNDING INFORMATION

This work was supported by the National Fish and Wildlife Foundation (NFWF) under grants #66509 and #70713.

## CONFLICT OF INTEREST STATEMENT

The authors have no competing interests to declare.

## Data Availability

Due to the sensitive nature of endangered species data, datasets or shapefiles containing specific GPS locations are not publicly available. Datasets and R code used for habitat selection modeling are located in the following Dryad repository: https://doi.org/10.5061/dryad.5tb2rbpb3. Endangered species data and Suisun Marsh lidar data may be available from the authors on reasonable request.
